# Supplementary material for: Small Molecules Temporarily Induce Neuronal Features in Adult Canine Dermal Fibroblasts
Source: Int J Mol Sci. 2023 Oct 31;24(21):15804. doi: 10.3390/ijms242115804 (PMC10648228; doi:10.3390/ijms242115804)
Supplement: Supplementary file 1 [file ijms-24-15804-s001.zip › ijms-2650351-supplementary.pdf]

# Small molecules temporarily induce neuronal features in adult canine dermal fibroblasts

**Kiyotaka Arai [1,*], Fumiyo Saito [2], Masashi Miyazaki [1], Haruto Kushige [1], Yayoi Izu [3], Noritaka Maeta [1] and Kazuaki Yamazoe [1]**

[1] Department of Veterinary Surgery, Faculty of Veterinary Medicine, Okayama University of Science, 1-3 Ikoi-no-oka, Imabari, 794-8555, Japan; k-arai@ous.ac.jp (K.A.); v18m131mm@ous.jp (M.M.); v19m067kh@ous.jp (H.K.); n-maeta@ous.ac.jp (N.M.); k-yamazoe@ous.ac.jp (K.Y.)
[2] Department of Toxicology, Faculty of Veterinary Medicine, Okayama University of Science, 1-3 Ikoi-no-oka, Imabari, 794-8555, Japan; f-saito@ous.ac.jp (F.S.)
[3] Department of Laboratory Animal Science, Faculty of Veterinary Medicine, Okayama University of Science, 1-3 Ikoi-no-oka, Imabari, 794-8555, Japan; y-izu@ous.ac.jp (Y.I.)
*****Correspondence: k-arai@ous.ac.jp (K.A.)

**Supplementary figure 1 (Figure S1)**

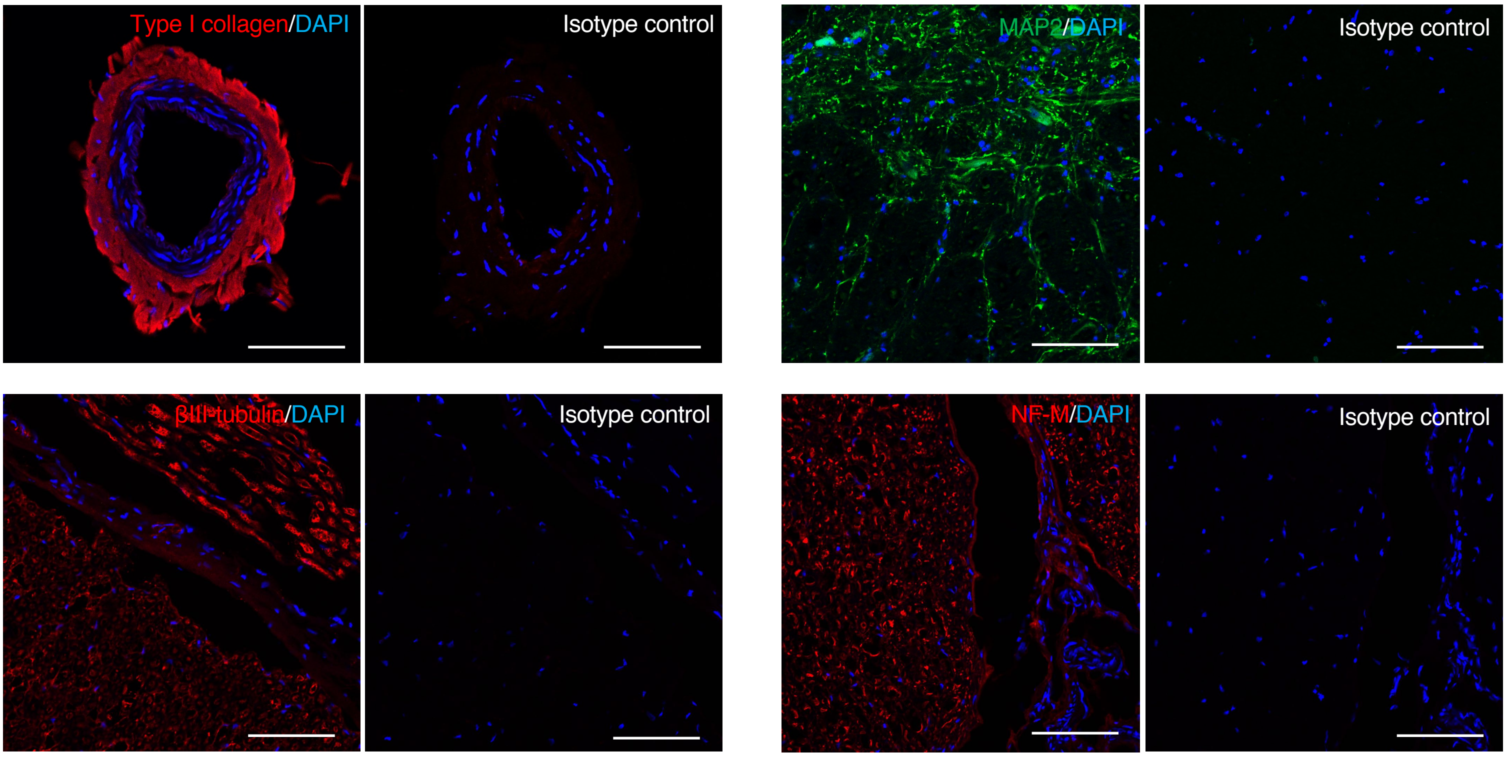


Supplementary figure 1 (Figure S1): Specific immunoreactivity of antibodies with canine antigens. Images represent the immunoreactivity of each antibody against canine histological intrinsic structures. Bars = 200 μm. The antibody against type I collagen selectively immunoreacts with the connective tissue of tunica adventitia of the spinal cord aorta. The antibodies against MAP2, βIII-tubulin, and neurofilament-medium selectively immunoreact with dendrites or axons, which constitute the neural structure of the spinal cord. Isotype controls do not show immunoreactivity .

**Supplementary figure 2 (Figure S2)**

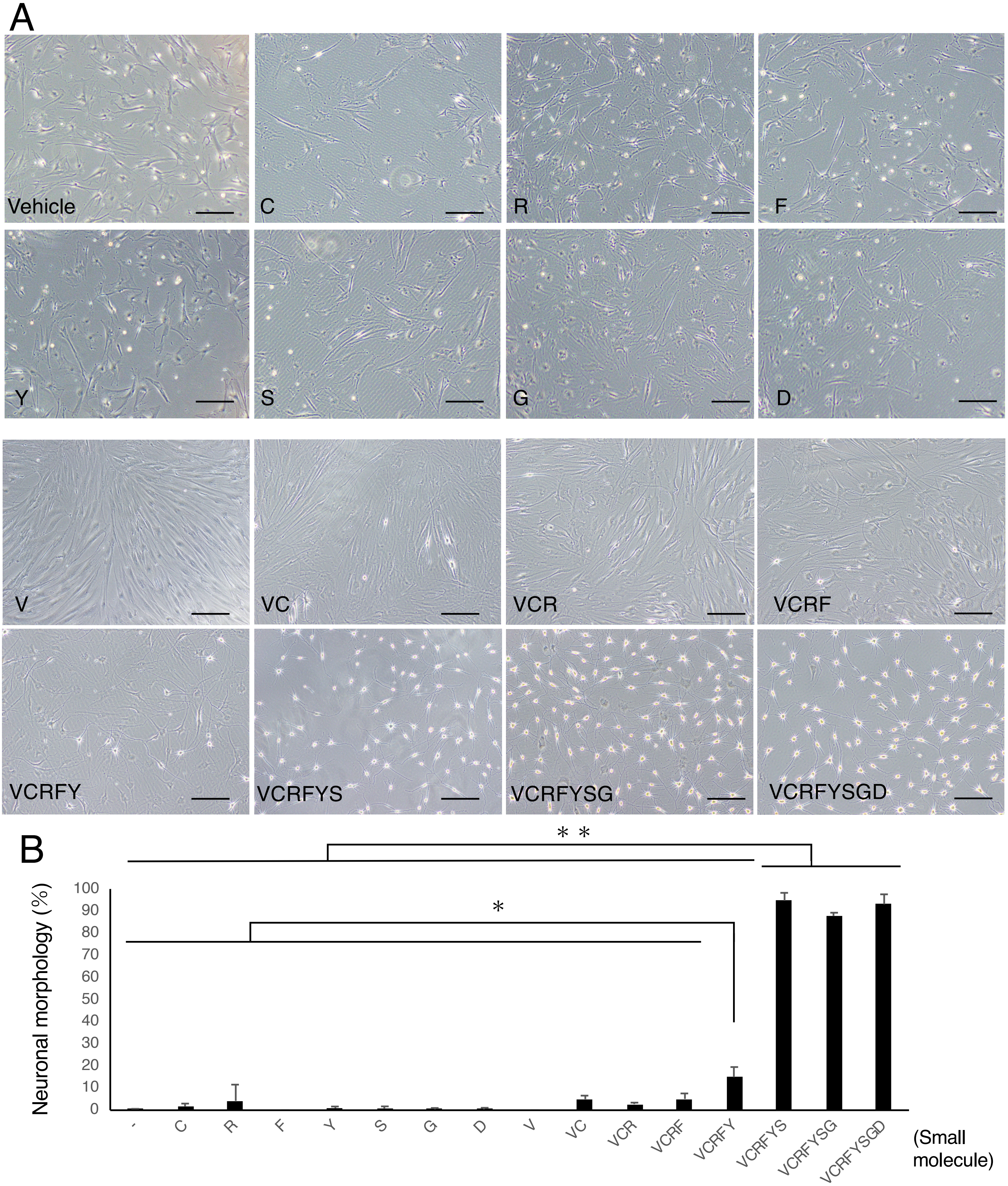


Supplementary figure 2 (Figure S2): Influence of a single small molecule (SM) or combination of SMs on neuronal morphological change after 6 days of the induction process. (A) A photomicrograph of adult canine dermal fibroblasts (ACDFs) treated with SMs (C, CHIR99021; R, Repsox; F, Forskolin; Y, Y-27632; S, SP600125; G, Go6983; D, dorsomorphin; and V, Valproic acid). Bar = 200 μm. (B) The proportions of ACDFs exhibiting neuronal morphology (*n* = 3). Any single SM has little impact on morphology (0–4.2%). However, some of the ACDFs change into neuronal morphology when treated with VCRFY (14.9%), and this is further increased significantly by the addition of SP600125, a c-Jun N-terminal kinase inhibitor (VCRFYS; 94.7%), Go6983, a protein kinase C inhibitor (VCRFYSG; 87.8%) and dorsomorphin, a bone morphogenetic protein/adenosine monophosphate kinase inhibitor (VCRFYSGD; 93.1%). *$p$ <0.01 and **$p$ <0.001.

**Supplementary figure 3 (Figure S3)**

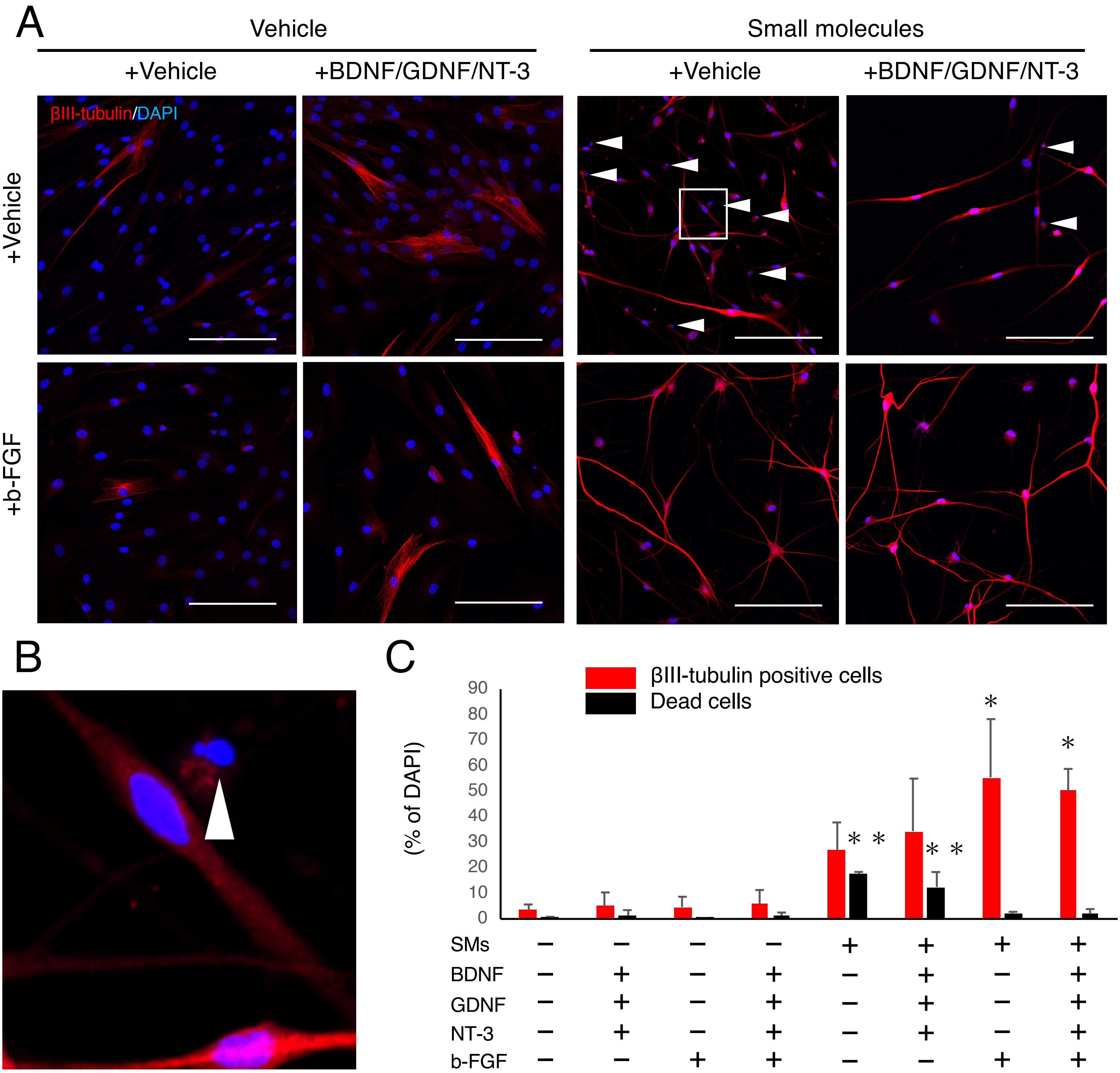


Supplementary figure 3 (Figure S3): Influence of cytokines on neuronal induction efficacy and cell death. (A) Fluorescent images of βIII-tubulin/4′,6-diamidino-2-phenylindole (DAPI). The images represent the immunoreactivity of βIII-tubulin in adult canine dermal fibroblasts (ACDFs) treated with the vehicle or the eight small molecules (SMs) in the presence of the vehicle only, brain-derived neurotrophic factor (BDNF)/glial cell line-derived neurotrophic factor (GDNF)/neurotrophin-3 (NT-3), basic fibroblast growth factor (b-FGF), or BDNF/GDNF/NT-3/b-FGF, BDNF/GDNF/NT-3, b-FGF, and BDNF/GDNF/NT-3/b-FGF (bars = 100 μm). (B) The white square of panel A shows an image of dead cells with nuclear condensation and fragmentation (arrowhead). (C) The proportions of βIII-tubulin-positive cells and dead cells are compared ($n = 3$). The proportions of βIII-tubulin-positive cells in the presence of the vehicle, BDNF/GDNF/NT-3, b-FGF, and BDNF/GDNF/NT-3/b-FGF are 3.5%, 5.1%, 4.7%, and 5.9%, respectively, in control groups treated without SMs, and 27.1%, 34.4%, 55.4%, and 50.1%, respectively, in SM-treated groups. βIII-tubulin-positive cells are significantly increased in the two SM groups treated in the presence of b-FGF and BDNF/GDNF/NT-3/b-FGF compared to the four groups without SMs. The proportions of dead cells in the presence of vehicle, BDNF/GDNF/NT-3, b-FGF, and BDNF/GDNF/NT-3/b-FGF are 0.4%, 1.3%, 0.1%, and 1.3%, respectively, in control groups treated without SMs, and 17.7%, 12.5%, 2.0%, and 1.9%, respectively, in SM-treated groups. The proportions of dead cells are significantly increased in two SM groups treated in the presence of the vehicle only and BDNF/GDNF/NT-3 compared to the other six groups. *$p < 0.01$ and **$p < 0.001$.

**Supplementary figure 4 (Figure S4)**

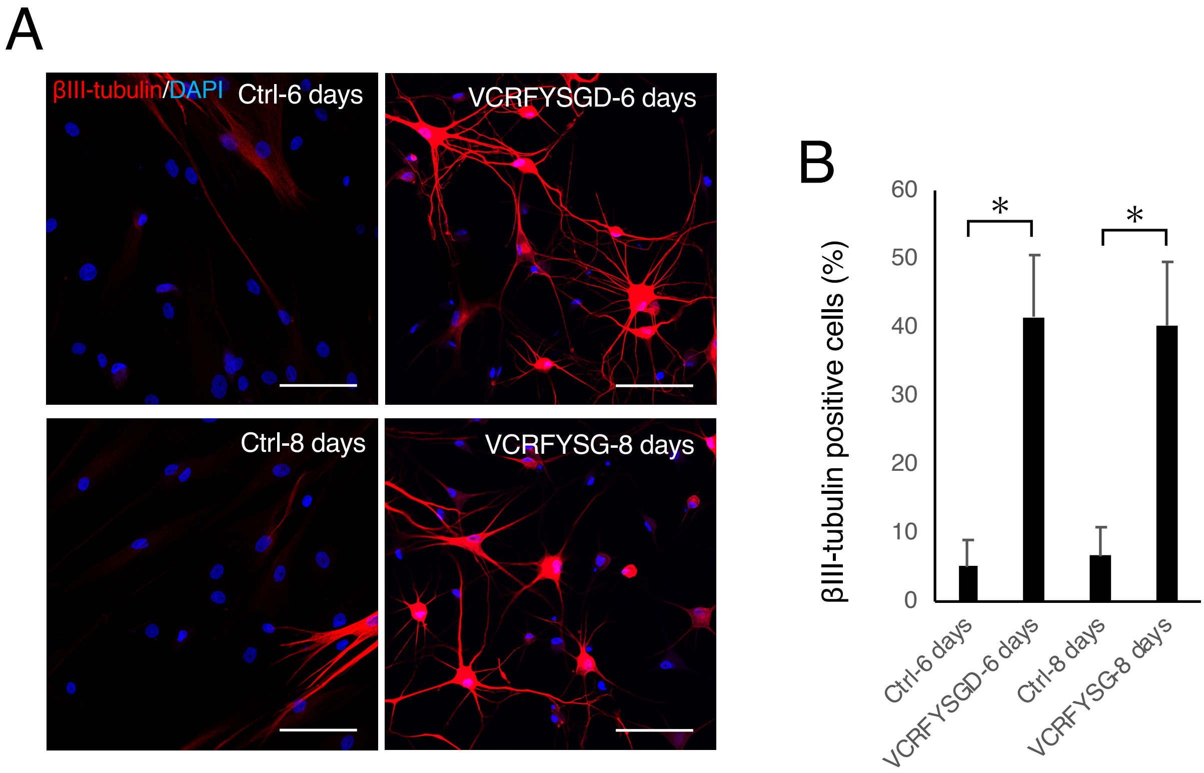


Supplementary figure 4 (Figure S4): Comparison of neuronal induction efficacy between our protocol, VCRFYSGD (V, Valproic acid; C, CHIR99021; R, Repsox; F, Forskolin; Y, Y-27632; S, SP600125; G, Go6983; D, dorsomorphin) for 6 days, and the original human protocol, VCRFYSG for 8 days [6]. After the induction process, the same maturation process [6] was conducted for 6 days. (A) Fluorescent images of βIII-tubulin/4′,6-diamidino-2-phenylindole (DAPI). The images represent the immunoreactivity of βIII-tubulin in adult canine dermal fibroblasts (ACDFs) treated with VCRFYSGD for 6 days or VCRFYSG for 8 days. Bar = 100 μm. ACDFs show neuronal morphology and exhibit immunoreactivity for βIII-tubulin in both protocols. (B) The proportions of βIII-tubulin-positive cells are almost the same in our protocol (41.6%) compared to the original human protocol (40.3%), with no significant differences. In both protocols, the proportions of small molecule-treated ACDFs are significantly increased compared to vehicle-treated control (Ctrl) cells ($n = 3$). *$p$ <0.01.
